# The emergence of superstructural order in insulin amyloid fibrils upon multiple rounds of self-seeding

**DOI:** 10.1038/srep32022

**Published:** 2016-08-25

**Authors:** Weronika Surmacz-Chwedoruk, Viktoria Babenko, Robert Dec, Piotr Szymczak, Wojciech Dzwolak

**Affiliations:** 1 Institute of High Pressure Physics, Polish Academy of Sciences, Sokolowska 29/37, 01-142 Warsaw, Poland; 2 Institute of Biotechnology and Antibiotics, Staroscinska 5, 02-516 Warsaw, Poland; 3 Department of Chemistry, Biological and Chemical Research Centre, University of Warsaw, Pasteura 1, 02-093 Warsaw, Poland; 4 Institute of Theoretical Physics, Faculty of Physics, University of Warsaw, Pasteura 5, 02-093 Warsaw, Poland

## Abstract

Typically, elongation of an amyloid fibril entails passing conformational details of the mother seed to daughter generations of fibrils with high fidelity. There are, however, several factors that can potentially prevent such transgenerational structural imprinting from perpetuating, for example heterogeneity of mother seeds or so-called *conformational switching*. Here, we examine phenotypic persistence of bovine insulin amyloid ([BI]) upon multiple rounds of self-seeding under quiescent conditions. According to infrared spectroscopy, with the following passages of homologous seeding, daughter fibrils gradually depart from the mother seed’s spectral characteristics. We note that this transgenerational structural drift in [BI] amyloid leads toward fibrils with infrared, chiroptical, and morphological traits similar to those of the superstructural variant of fibrils which normally forms upon strong agitation of insulin solutions. However, in contrast to agitation-induced insulin amyloid, the superstructural assemblies of daughter fibrils isolated through self-seeding are sonication-resistant. Our results suggest that formation of single amyloid fibrils is not a dead-end of the amyloidogenic self-assembly. Instead, the process appears to continue toward the self-assembly of higher-order structures although on longer time-scales. From this perspective, the fast agitation-induced aggregation of insulin appears to be a shortcut to amyloid superstructures whose formation under quiescent conditions is slow.

The *in vivo* role of amyloid fibrils, whether pathogenic or biologically-functional^1^,^2^,^3^, is intimately connected to their 3D-structures which, in contrast to the globular precursors, are not unambiguously encoded in the amino acid sequences. Currently, polymorphism of amyloid fibrils is a well-recognized phenomenon^4^,^5^,^6^,^7^,^8^,^9^,^10^. Its thermodynamic origins are rooted in energy landscapes of amyloid fibrils which do not enforce one particular mode of self-association of β-strands but instead are permissive to many distinct structural variants of aggregates^5^,^11^,^12^,^13^. The manifold of energetically-accessible modes of amyloidogenic self-assembly and the autocatalytic manner of their structure-templating proliferation are the key non-Anfinsenian aspects of protein fibrillation. The interplay of these two factors manifests in the phenomenon of *self-propagating polymorphism* of amyloid fibrils wherein structural phenotype of the mother seed is spread through autocatalytic loops to large quantities of the misfolded protein. Such self-propagating variants of fibrils assembled from covalently identical polypeptide chains are termed *strains* by the analogy to proliferation patterns of mammalian “*prion strains*” – distinct forms of PrP-amyloid-related Creutzfeldt-Jakob disease observed in individuals with expression of identical precursor PrP^C^ protein^14^,^15^,^16^,^17^,^18^. The same type of conformational polymorphism is thought to underlie the existence of *strains* among yeast prions^19^,^20^,^21^.

The essential features of the strain propagation can be reproduced *in vitro* using amyloidogenic proteins and peptides such as insulin^22^,^23^,^24^,^25^, K3 fragment of β_2_-microglobulin^26^, glucagon^27^, fragments of tau protein^28^, and Aβ-peptides^29^,^30^. Transgenerational stability of an amyloid phenotype can be decreased by heterogeneity of mother seed samples (e.g. refs 28, 31), and possibly microscopic-scale events compromising fidelity of the conformational imprinting such as secondary nucleation and conformational switching^32^.

Insulin is often employed as an insightful *in vitro* model to study mechanisms of amyloidogenic self-assembly in general^33^,^34^,^35^,^36^, as well as its particular aspects such as the self-propagating polymorphism^22^,^23^,^24^,^25^, or formation of highly-ordered amyloid superstructures^37^,^38^,^39^,^40^. Here, our primary interest was to study persistence of the phenotype of [BI] amyloid formed spontaneously at 65 °C and under quiescent conditions upon multiple rounds of homologous seeding to BI solutions at 37 °C where *de novo* nucleation is significantly decelerated compared to seed-induced aggregation. Swift evolution of following generations of [BI] amyloid visible on the conformational and morphological levels implies instability of the *de novo* formed phenotype. Our findings also suggest that formation of single amyloid fibrils is not the end of the amyloidogenic self-assembly which continues towards higher-order structures, albeit on longer time-scales.

## Results and Discussion

**Figure 1:**
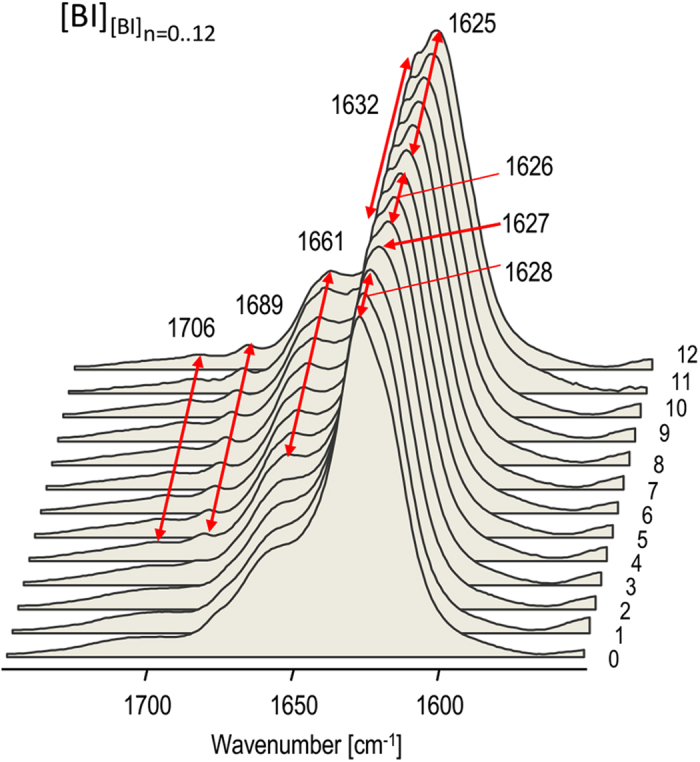
Persistence of fingerprint infrared features (amide I’ band region) characteristic for [BI] amyloid upon repeated passages of homologous seeding. Mother [BI] amyloid (marked as 0^th^ generation) was obtained through spontaneous quiescent aggregation of 1 wt. % BI in 0.1 M NaCl in D_2_O, pD 1.9 at 65 °C. Subsequently, amyloid fibrils of nth-generation were used to induce (n + 1)th generation of fibrils (indicated on the right side) through 96 h-long quiescent incubation at 37 °C of 1 wt % native BI in 0.1 M NaCl, D_2_O, pD 1.9 in the presence of preformed sonicated seeds (1:100 mass ratio of amyloid: native BI). Samples of grown daughter fibrils were sonicated prior to FT-IR measurements in order to sharpen spectral features.

We have employed Fourier transform infrared (FT-IR) spectroscopy as a high-throughput probe of stability of [BI] structural phenotype upon multiple rounds of self-seeding. The vibrational amide I’ band is not only informative of the conformation of protein backbone but is also sensitive to fine features of spatial arrangements of aggregated β-strands (β-sheet twists, strength of inter-strand hydrogen bonds, etc.), hence different amyloid strains often reveal distinct fingerprint features in this spectral region (e.g. refs 23, 41). Figure 1 shows stacked infrared spectra of mother [BI] amyloid and following twelve generations of [BI] fibrils induced by subsequent rounds of self-seeding. These experiments were carried out under conditions disfavoring *de novo* formation of competitive amyloid nuclei (i.e. at mild temperature and without agitation) and therefore promoting propagation of the seed’s phenotype. From the data shown in Fig. 1, it is clear that the first two generations of daughter fibrils are very similar in terms of infrared features to the mother amyloid as the amide I’ band remains firmly centered at 1628 cm^−1^. However, with the third generation ([BI]_[BI]3_), fibrils began to depart from the original template’s spectral characteristics: there is a minute red shift of the amide I’ band maximum to 1627 cm^−1^ followed by the splitting of the band into two peaks at 1626 cm^−1^ and 1632 cm^−1^ observed for the fourth, fifth, and sixth generations. These changes occur simultaneously with the emergence of three poorly resolved peaks in the high frequency region at 1661, 1689, and 1706 cm^−1^. For the seventh generation of fibrils, there is an additional red-shift of the main spectral component to 1625 cm^−1^ beyond which (up to the twelfth generation of daughter fibrils examined in this study) no further spectral drift is observed. Therefore we conclude that the new [BI]_[BI]12_ variant isolated through multiple rounds of self-seeding is stable regarding further transgenerational proliferation, at least under the seeding conditions employed in this study.

**Figure 2:**
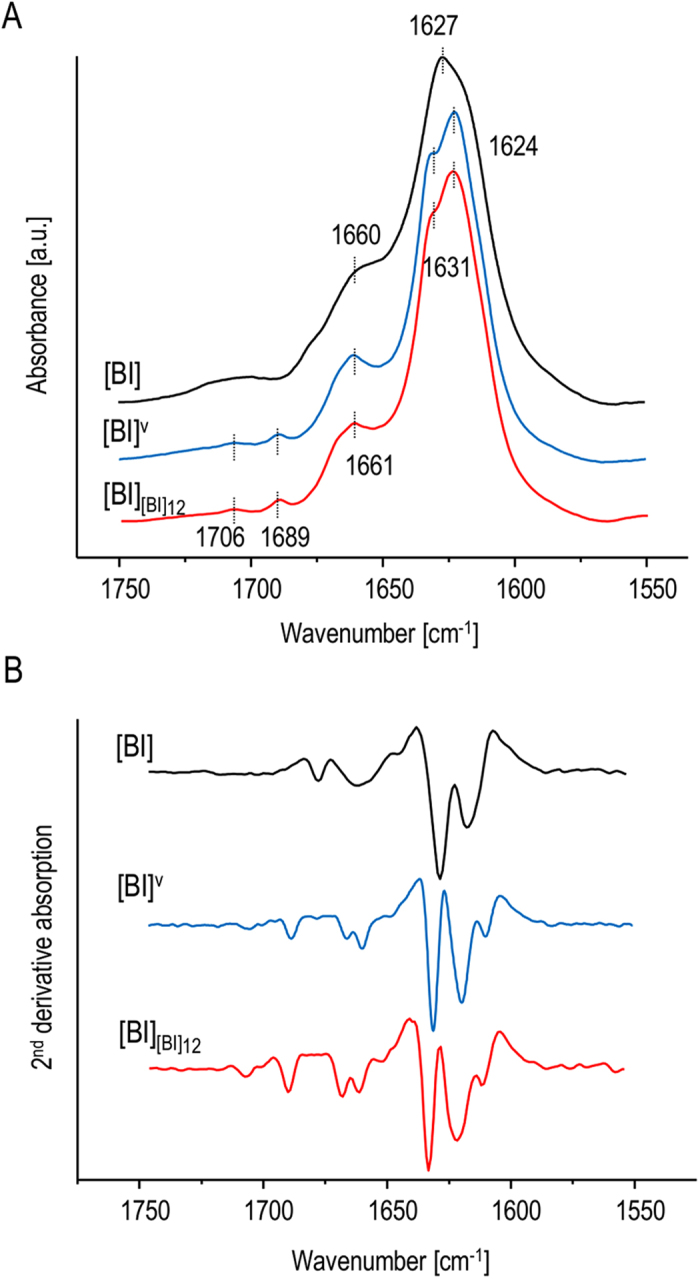
Comparison of FT-IR spectra of various insulin amyloid preparations. (**A**) Original absorption and (**B**) 2^nd^ derivative FT-IR spectra of [BI] fibrils obtained through agitation-assisted (blue) and quiescent (black) aggregation (no seeds added in either case), and of 12^th^ generation of [BI] amyloid induced by the homologous seeding as described in Fig. 1 (red).

Strikingly, the spectral features of [BI]_[BI]12_ amyloid, such as the characteristic splitting of the amide I’ band, are reminiscent of the particular superstructural variant of insulin fibrils known to form spontaneously upon intensive agitation of acidified bovine insulin solutions in the presence of sodium chloride^37^,^38^,^39^,^42^. Apart from the characteristic twisted morphology comprising of tightly aligned individual fibrils, its most distinct features are strong chiroptical properties manifesting in the capacity to induce either positive or negative extrinsic Cotton effect (or *induced circular dichroism*) in amyloid-specific achiral fluorophore - Thioflavin T (ThT)^37^,^38^,^39^ which is used routinely for amyloid detection due to its affinity to fibrils and powerful enhancement of fluorescence upon binding to fibrils^43^. For a comparison with infrared spectra of the twelfth-generation of self-seeded bovine insulin fibrils ([BI]_[BI]12_), chiral superstructures of insulin fibrils (labelled [BI]^V^) were prepared following the typical protocol involving intensive agitation of acidified BI solutions in 0.1 M NaCl at increased temperature^37^,^38^,^39^ (using the same sample and temperature conditions but without agitation results in the formation of regular [BI] fibrils described in Materials and Methods). In Fig. 2, the original absorption and second-derivative FT-IR spectra of thus obtained amyloid [BI]^V^ are juxtaposed with those of [BI]_[BI]12_ daughter fibrils and fibrils grown *de novo* under quiescent conditions ([BI]). We note the remarkable resemblance of fingerprint infrared traits of [BI]^V^ and [BI]_[BI]12_ also in terms of the fine high frequency peaks at 1706, 1689, and 1661 cm^−1^ which, along with the 1631/1624 cm^−1^ split of the main spectral component, clearly distinguish these amyloid specimen from [BI] fibrils. The high degree of similarity of [BI]^V^ and [BI]_[BI]12_ is confirmed after the enhancement of spectral features (Fig. 2b): very similar sets of peaks (both positive and negative) are observed in the computed second derivative spectra of [BI]^V^ and [BI]_[BI]12_, although there is a minor broadening of the negative peak at 1624 cm^−1^ for [BI]_[BI]12_ relative to [BI]^V^.

**Figure 3:**
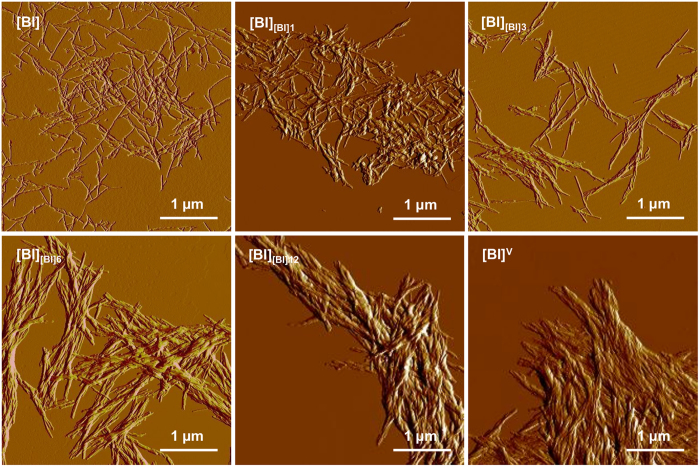
Morphological analysis of various insulin fibrils. Amplitude AFM images of non-sonicated insulin aggregates: mother [BI] amyloid (0^th^ generation), 1^st^, 3^rd^, 6^th^, 12^th^ generations of fibrils obtained through homologous seeding at 37 °C, and of agitation-induced [BI]^V^ amyloid (as indicated in panels).

As the infrared data shown in Figs 1 and 2 are symptomatic of a progressive structural drift of self-seeded insulin amyloid toward the chiral superstructures, we have carried out morphological and chiroptical characterization of selected generations of fibrils to verify this hypothesis. The peculiar morphology of chiral insulin amyloid superstructures typically precludes precise atomic force microscopy (AFM) - based measurements of individual fibrils (e.g. in terms of diameter etc. refs 38, 39). Hence the morphological characterization was limited to assessment of the degree of lateral association and superstructural order. In Fig. 3, amplitude AFM images of insulin fibrils grown under quiescent conditions – either *de novo* ([BI]) or through self-seeding ([BI]_[BI]1_, [BI]_[BI]3_, [BI]_[BI]6_, [BI]_[BI]12_) are compared with that of [BI]^V^. Typically, mother [BI] amyloid specimen are singly dispersed and the lateral association of individual fibrils is limited. However, for the following generations of seeded amyloid the degree of self-association gradually increases. For the third and sixth generations, the laterally aligned fibrils become twisted. The increasing tendency to align laterally and form higher order entities is also evident for the sixth and twelfth generations of daughter amyloid. Eventually, the twelfth generation of fibrils self-assemble into a tightly packed and highly ordered matrix with the appearance very similar to that of agitation-induced [BI]^V^ amyloid.

**Figure 4:**
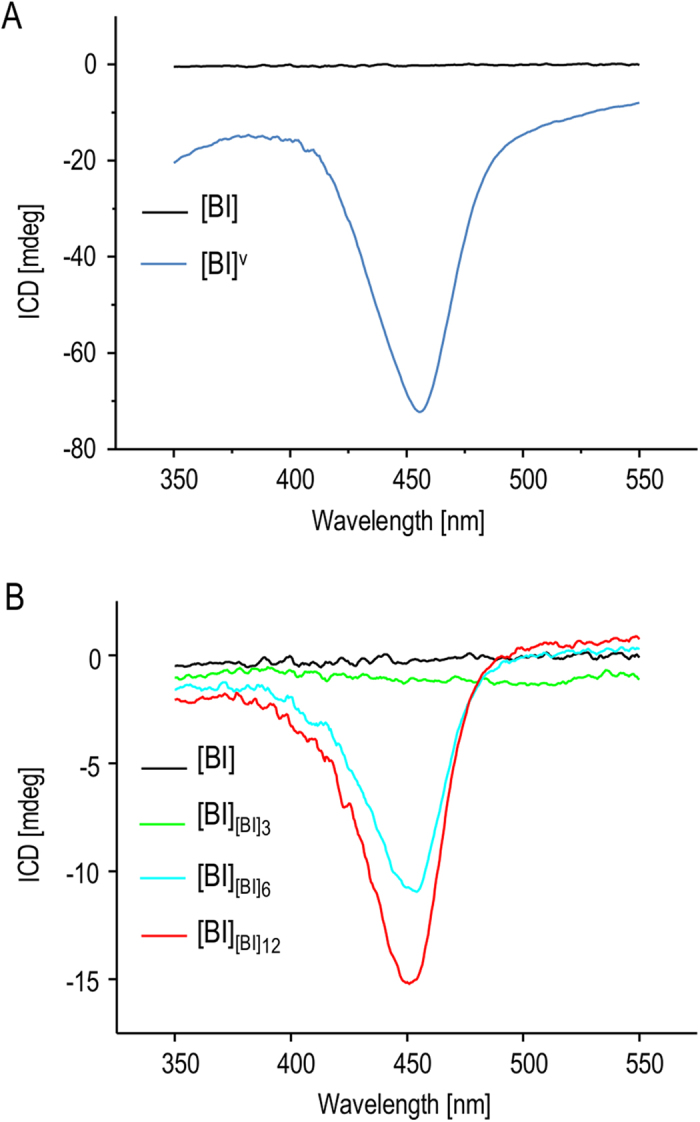
Chiroptical properties of insulin amyloid fibrils. (**A**) ICD spectra of ThT-stained [BI] (black) and [BI]^V^ fibrils (blue). (**B**) ICD spectra of ThT-stained daughter fibrils (0^th^, 3^rd^, 6^th^, and 12^th^ generations) obtained through quiescent aggregation in the presence of seeds at 37 °C.

At this point, it became crucial to confirm whether the observed morphological evolution of self-seeded [BI] towards chiral superstructures is reflected in changes of chiroptical properties. Figure 4a shows induced circular dichroism (ICD) spectra of ThT-stained [BI] and [BI]^V^ fibrils. In accordance with previous studies, only agitation-induced amyloid ([BI]^V^) is capable of imprinting its net chiral bias in bound achiral ThT resulting in the electronic transition of the dye becoming CD-active^44^. The negative sign of the induced circular dichroism is typical for superstructural chiral insulin amyloid assemblies (so-called “−*ICD amyloid*”) precipitating from agitated insulin solutions above 50 °C^37^,^38^,^39^. The ICD spectra shown in Fig. 4B correspond to ThT-stained daughter generations of fibrils morphologically characterized in Fig. 3. It is clear that [BI]_[BI]6_ and [BI]_[BI]12_ generations previously shown to form superstructural patchworks also induce negative extrinsic Cotton effects in ThT molecules although with intensities roughly five times lower than of the ThT complex with agitation-induced [BI]^V^ amyloid.

**Figure 5:**
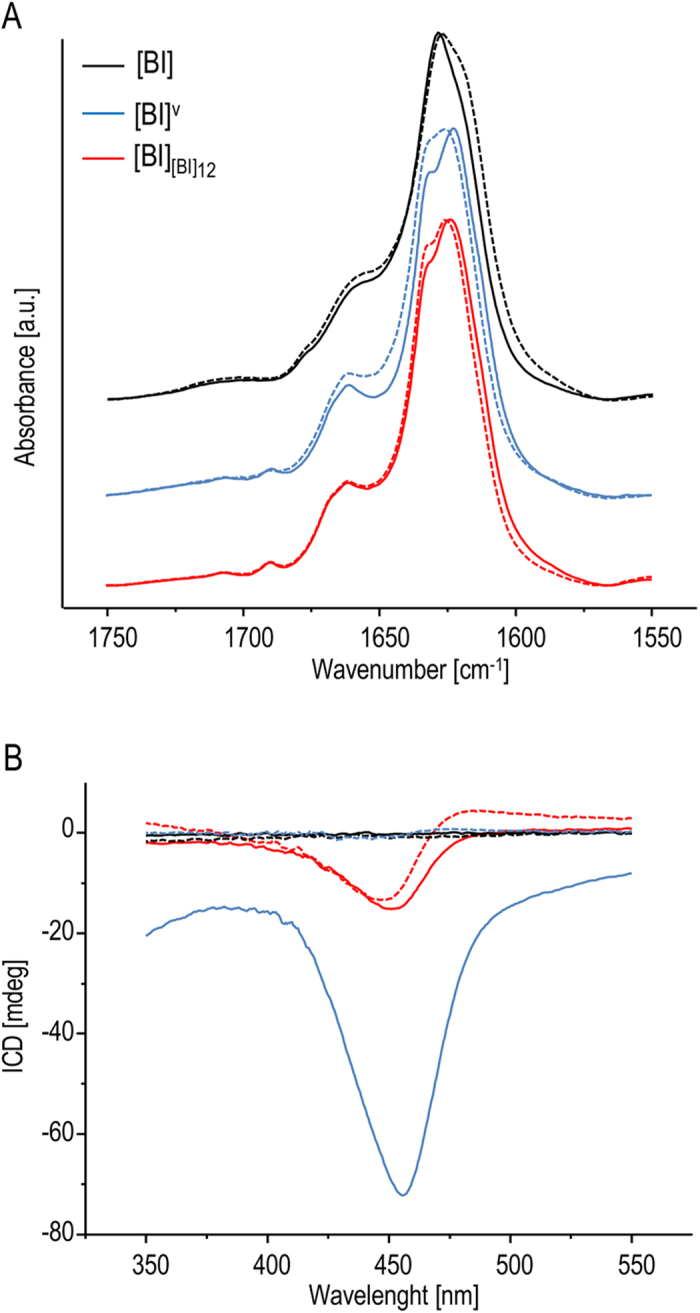
Effects of sonication on various insulin fibrils. FT-IR (**A**) and ThT-ICD (**B**) spectra of insulin fibrils formed spontaneously without seeds and after multiple self-seeding rounds (12^th^ generation of [BI] – red). Solid lines correspond to spectra before sonication whereas dashed lines mark spectra of sonicated samples.

The microscopic and spectroscopic data presented so far strongly suggest that multiple rounds of self-seeding of [BI] fibrils lead to the emergence of a structural variant of bovine insulin amyloid very similar to the one forming spontaneously when the precursor insulin solution is subjected to vigorous agitation. One puzzling aspect of this conclusion is that sonication has been explicitly shown to disassemble such amyloid superstructures^38^ whereas precisely this treatment is repeatedly employed in this study to every generation of fibrils before using them as seeds. Hence, it appears that a mechanism of accumulation of sonication-resistant amyloid superstructures is involved in the transgenerational transfer of the structural blueprint. Figure 5 depicts the effects of sonication on FT-IR and ICD spectra of [BI], [BI]^V^, and [BI]_[BI]12_ fibrils. For all the structural variants of insulin amyloid examined here, infrared spectra of [BI]_[BI]12_ are least affected by the 60 s-long ultrasound treatment. There is a minor increase in intensity of the 1632 cm^−1^ component but no other significant changes in amide I’ bandwidth or spectral/intensity shifts above 1650 cm^−1^ are observed. This contrasts with more pronounced changes that are induced by sonication of [BI]^V^ with the two highest intensity components becoming less resolved and partly overlapping while the 1661 cm^−1^ peak becomes more diffuse. According to the ensuing ICD data, the variation in susceptibility to sonication between [BI]^V^ and [BI]_[BI]12_ is more dramatic in regard to their chiroptical properties. The brief ultrasound treatment leads to virtual disappearance of the extrinsic Cotton effect in [BI]^V^-bound ThT, yet exerts only a limited impact on the ThT-[BI]_[BI]12_ complex. This implies a higher degree of mechanical stability of chiral superstructures formed gradually through multiple seeding rounds than of those formed rapidly by application of vortex forces. We have also compared the effects of sonication on morphology of [BI]^V^ and [BI]_[BI]12_ using AFM (Supplementary Information). While the ultrasound-induced disassembly of [BI]^V^ produces small scattered particles, [BI]_[BI]12_ appears to be more resistant to such treatment: amyloid superstructures do break down but the remaining aggregates are clump-like assemblies. Hence the AFM data parallels the distinct effects sonication has on infrared and ICD spectra of [BI]^V^ and [BI]_[BI]12_.

**Figure 6:**
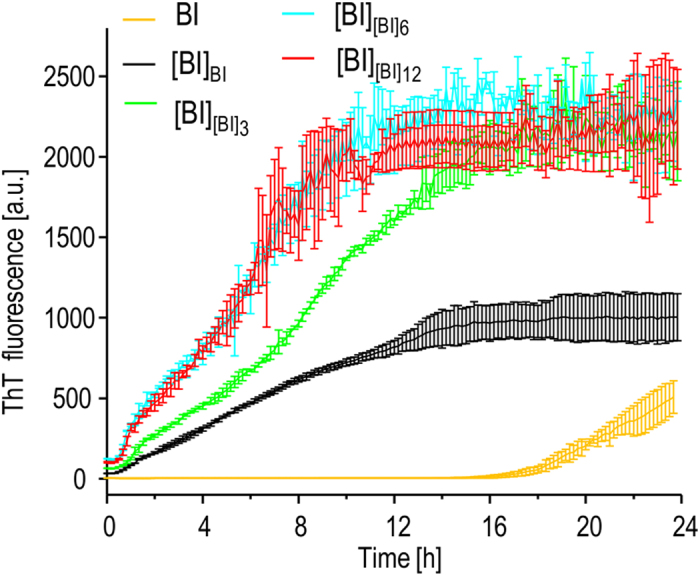
ThT-fluorescence-probed kinetics of bovine insulin fibrillation under different seeding conditions. Trajectories measured in the absence of seeds are marked with yellow line, and for samples seeded at 37 °C with sonicated selected generations of daughter [BI] are marked with specified colors. Error bars correspond to standard deviations calculated for several independently obtained kinetic trajectories.

While infrared spectra capture secondary structure-level aspects of the transgenerational drift in [BI] fibrils, AFM imaging and ICD spectroscopy report on changes in higher-order organization of aggregates. The emergence of sonication-resistant structural variant of [BI] amyloid with the tendency to form tightly-aligned fibrils could result in reduction of fibril tips: amyloid mass ratio in daughter templates (which, as larger objects, could also disperse in the seeded solution less effectively). This, in turn, could cause a significant deceleration of fibrillation in subsequent seeding rounds. We have addressed this problem by examining kinetics of insulin aggregation induced by different generations of self-seeded [BI]. Aggregation kinetics were probed using plate reader coupled to the standard ThT-based fluorescence assay. Averaged trajectories of ThT fluorescence intensity of BI solutions seeded with different freshly sonicated amyloid templates are shown in Fig. 6. Under the conditions of these measurements, and in the absence of any seeds, the process of *de novo* insulin fibrillation is rather slow with approximately 16 h-long lag-phase. In the presence of sonicated mother [BI] amyloid, fibrillation starts immediately without any detectable lag-time and the whole transition is complete within 14 hours. When the original [BI] templates are replaced with third, sixth and twelfth generations of amyloid the fibrillation is accelerated (for seeding with [BI]_[BI]6_ and [BI]_[BI]12_ the aggregation takes roughly 10 hours). The considerably higher final ThT fluorescence plateaus for insulin seeded with daughter fibrils are likely to originate from differences between [BI] and [BI]_[BI]6_/[BI]_[BI]12_ in: [i] number of accessible ThT-binding sites per amyloid mass unit, [ii] corresponding ThT-binding constants, and [iii] individual quantum yields of ThT molecules bound to different amyloid specimen^45^. More importantly, the data in Fig. 6 shows clearly that the transgenerational drift to the superstructural amyloid variant does not decrease the catalytic potential of daughter seeds.

**Figure 7:**
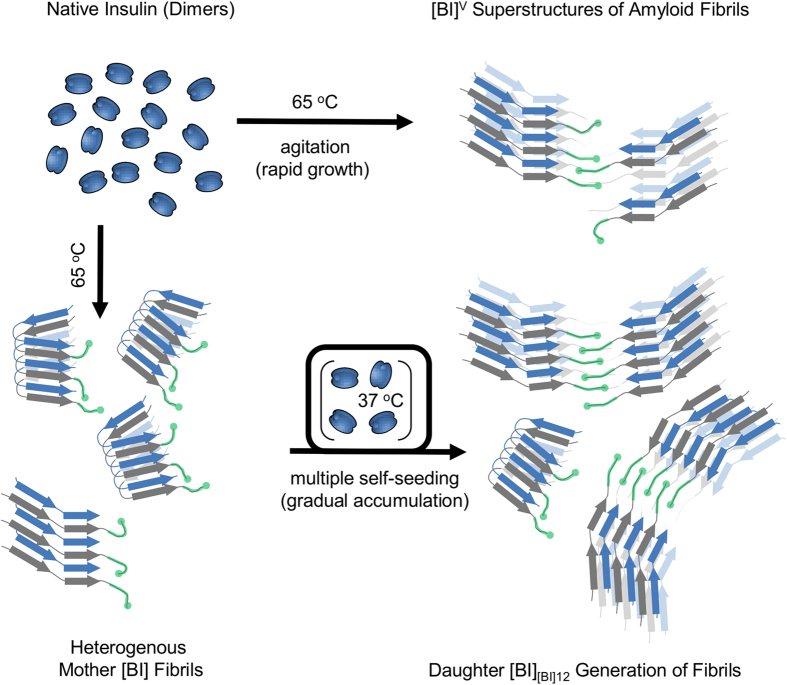
Two different pathways of the self-assembly of chiral superstructures of insulin amyloid fibrils. Chiral superstructures of insulin amyloid fibrils ([BI]^V^ – marked as “open” stacks of beta-sheets composed of alternate blue and grey strands corresponding to A and B chains of insulin) form rapidly upon intensive agitation of heated solutions of dimeric bovine insulin. Tiny amounts of [BI]^V^-like structures are likely to form also during insulin fibrillation under quiescent conditions when regular singly dispersed [BI] fibrils (marked as “folded” stacks of beta-sheets) are predominantly formed. Since different elongation rates of [BI]^V^ and [BI] favor the former phenotype upon multiple rounds of self-seeding under quiescent conditions, chiral superstructures of insulin amyloid (presumably bound together by B-chain C-termini – marked in green color) dominate in the following generations of daughter fibrils. The rapid growth of agitation-induced fibrils is likely to result in high concentrations of structural defects rendering thus formed superstructures less rigid and more vulnerable to sonication compared to the chiral variants slowly accumulating upon repeated seeding.

Results of this study indicate that populations of structural variants of fibrils that dominate in samples of spontaneously formed amyloid may be unstable upon multiple rounds of self-seeding. It is unclear through which mechanism the [BI]^V^-like amyloid emerges as the winning phenotype. In principle, there is possibility of so-called *conformational switching* occurring spontaneously within a growing fibril: a self-propagating structural change taking place during the elongation. This phenomenon has been reported upon cross-seeding of mouse PrP monomers with preformed hamster prion seeds (S-strain). Because of differences in the primary structures, mouse PrP lacks the capacity to adopt the exact conformation and packing patterns of the hamster S-fibrils which results in the recruited mouse PrP acquiring an alternative R-strain conformation^32^. In the case of fibrils built of covalently identical protein units, as studied in this paper, factors promoting switching are less obvious but still feasible (for example the lowest Gibbs energy variants of insulin amyloid at 65 °C and 37 °C could, hypothetically, be different). It seems, however, that in the case of multiple rounds of self-seeding, such scenario is unlikely. The routine of repetitive sonication of subsequent generations of daughter fibrils prior to using them as seeds has two important consequences. First, it multiplies concentration of catalytically active tips. Second, it also decreases any statistical impact of possible conformational switching events. We have created an auxiliary mathematical model to study how periodic sonication suppresses effects of spontaneous conformational switching (reversible or not) on proliferation of amyloid strains (Supplementary Information). Our argument based on that model is that even if conformational switching does take place, the cyclic fragmentation of seeds through sonication would slow down proliferation of the “switched” conformation in daughter generations of fibrils dramatically. In light of our analysis, it is unlikely that the swift transition from [BI] to [BI]^V^-like phenotype occurring (in spite of the periodic ultrasound treatments) in fibrils transitioning through the second, third, and fourth generations (Fig. 1) is caused by conformational switching. On the other hand, the data shown in Fig. 6 suggests that the alternative [BI]^V^-like phenotype may have a kinetic advantage in proliferation through elongation over [BI]. Hence, a more conventional explanation based on the assumption that a small fraction of [BI]^V^-like strain is formed already during quiescent *de novo* aggregation, only to spread later upon “self-seeding”, might be explored. In our model (visualized in Fig. 7), the basic distinction between the two strains begins on the level of secondary structure and is likely to involve different locking modes of the steric zippers within insulin amyloid core^46^. This assumption is in agreement with the fact that the secondary-structure sensitive amide I’ band varies between the two amyloid strains. The different chiroptical and morphological properties are the consequences of the conformational dimorphism transferred to higher levels of structural assembly (Fig. 7).

The main kinetic distinction between [BI] and [BI]^V^-like variants may be that nucleation of the former and elongation of the latter are the fastest accessible amyloidogenic routes. We have built a numerical model describing the conformational [BI] → [BI]^V^ drift upon multiple rounds of self-seeding based on such an assumption. Essentially, the same mechanism has been confirmed and successfully employed in preparation of structurally homogenous Aβ_1-40_ fibrils^31^. Also, very recently similar scenario was implicated in the structural drift taking place upon self-seeding of fibrils from K18 construct of tau protein^28^. Furthermore, one could hypothesize that fast *de novo* formation of [BI]^V^ upon agitation of insulin solution^37^,^38^,^39^ can be explained in a similar fashion with fragmentation of fibrils achieved not by rounds of sonication but by applying turbulent hydrodynamic forces. In the latter case, the rapid growth of agitation-induced fibrils ([BI]^V^) could result in high concentration of structural defects (especially so-called “inclusions”^47^) rendering the amyloid less rigid and more vulnerable to sonication compared to the chiral variant gradually accumulating upon repeated self-seeding ([BI]_[BI]12_). Such scenario could, in principle, account for the marked differences in response to ultrasound treatment reflected in the data in Fig. 5 and in Supplementary Information. In other words, despite the varied resistance to sonication, we may speculate that very similar motifs of β-strand stacking and spatial superstructural organization of protofilaments are present in molecular architectures of both [BI]_[BI]12_ and [BI]^V^ with the mechanical properties of the latter being compromised due to structural defects on the mesoscopic level. It should be stressed that presently, in the absence of definite high resolution structural data, the above reasoning remains only a working hypothesis for follow-up studies to be undertaken.

One puzzling finding presented in this study concerns the fact that daughter amyloid generations, while gradually drifting toward superstructures, when used as seeds, act as increasingly effective catalysts (Fig. 6). One could hypothesize that insulin B-chain’s C-terminal fragments, which are thought to protrude freely from single insulin fibrils^48^ but become inaccessible in [BI]^V^ superstructures stabilizing them as molecular Velcro^49^, are playing a role in this behavior. These fragments could transiently interact with aggregation-competent insulin monomers after dissociation of dimers, but prior to docking at the fibrils ends. Such interactions which are likely only in the case of seeds consisting of single fibrils could effectively slow down the elongation process compared to [BI]^V^ seeds. Certainly, further studies are needed in order to verify plausibility of this scenario.

**Figure 8:**
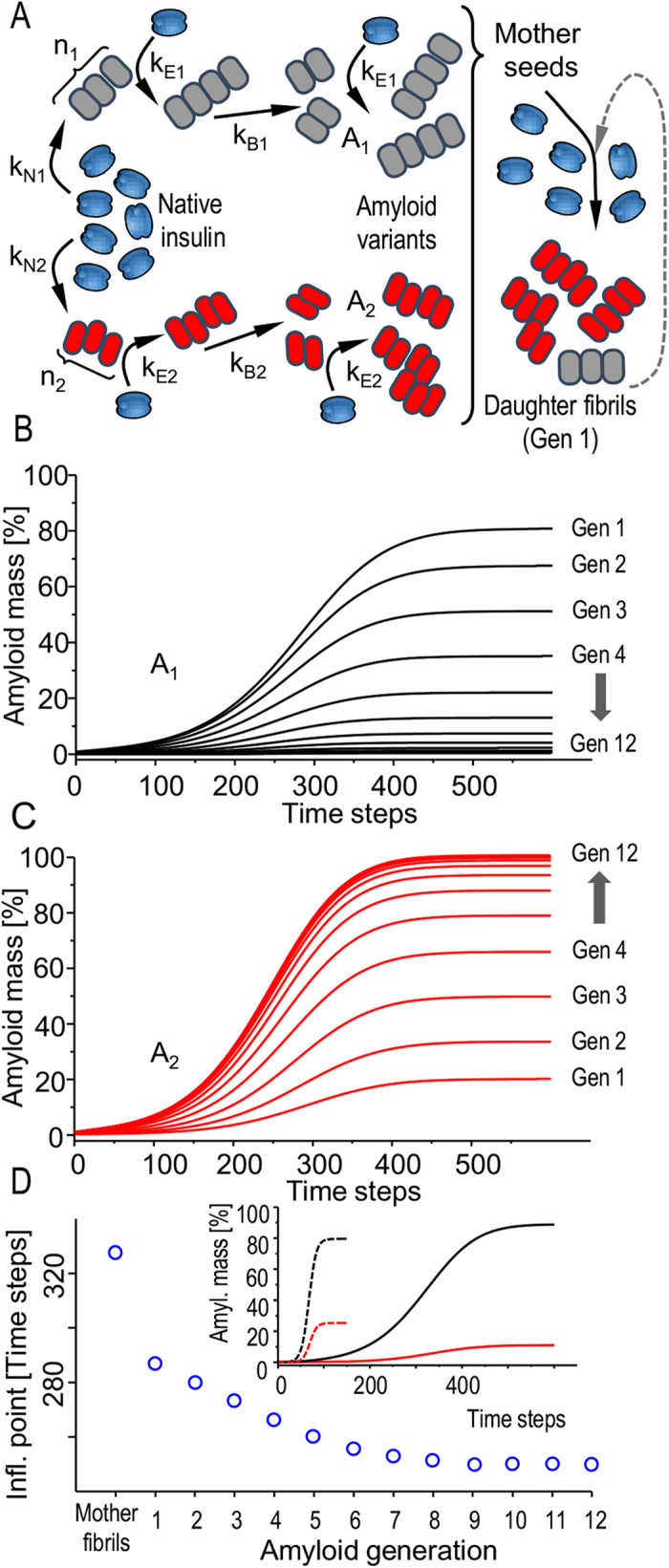
Simulation of competitive proliferation of two amyloid strains A_1_ and A_2_ upon subsequent rounds of self-seeding. (**A**) Model presumptions: two alternative structural variants of insulin fibrils form *de novo*. In general, different sets of kinetic parameters such as nuclea size (n), nucleation (k_N_), elongation (k_E_), and fragmentation rates (k_B_) are considered for both strains. A_1_ strain has a kinetic advantage (higher k_N_) over A_2_ and therefore dominates in the *de novo* formed mother fibrils which are subsequently used to induce daughter generation of fibrils with a similar set of constrains. The seeding is repeated for the 12 following generations of fibrils. Calculated sigmoidal curves of growth of populations of A_1_ (**B**) and A_2_ (**C**) triggered by following generations of daughter seeds show the increasing ratio of fast-elongating A_2_ fibrils. (**D**) Calculated deflection points of transition curves (total amyloid mass vs. time) for the following rounds of self-seeding. In the inset, *de novo* transition curves for quiescent (solid lines) and agitated (dashed lines) conditions are compared (black for A_1_ and red for A_2_).

Figure 8 presents results of numerical simulations of growth of two competing amyloid variants (A_1_ and A_2_) based on the kinetic model of prion propagation introduced by Collins *et al.*^50^. For sets of variable parameters such as rates of fibril nucleation, elongation and fragmentation (Fig. 8A, Materials and Methods) separately defined for each strain we can clearly observe correspondence with the main results of this study. Namely, population of the fast-nucleating strain (A_1_) is dominant in heterogeneous mother fibrils formed under quiescent conditions. However, with the following routes of self-seeding, the fast-elongating A_2_ strain gradually replaces A_1_ in daughter generations of fibrils (Fig. 8B,C). It is worth noting that as a consequence, the subsequent seeding rounds accelerate (vide deflection points of the overall kinetic curves in Fig. 8D) which is in accordance with the experimental data in Fig. 6. Although this model does not explicitly include processes of lateral aggregation and secondary nucleation which could play a role in the emergence of [BI]^V^, it does capture the increase of A_2_ variant ratio in mother fibrils when the *de novo* aggregation is carried out under turbulent conditions represented by increased rates of fibril fragmentation (inset in Fig. 8D).

In summary, our study sheds light on the complex problems of polymorphism and multiple transition pathways in insulin amyloid fibrils. We have demonstrated “populational” metastability of single bovine insulin fibrils formed spontaneously relative to the superstructural amyloid variant that self-assembles when agitation is applied during insulin aggregation. Our findings highlight the existence of slow structural dynamics involving whole amyloid fibrils on the way toward larger assemblies. Given the intricate nature of the underlying processes, further work is needed to explain relationship between the “populational” and thermodynamic metastabilities of amyloid fibrils and to answer the question of *What is the actual dead-end product of amyloidogenesis?*

## Materials and Methods

### Samples

BI was from Sigma-Aldrich (USA), D_2_O (“99.8 atom % D” grade) was from ARMAR Chemicals, Switzerland, and deuterium chloride (35 wt. % DCl solution in D_2_O, 99 atom % D) was from Sigma-Aldrich.

Insulin amyloid fibrils were obtained through incubation of freshly-prepared 1 wt. % BI in 0.1 M NaCl in D_2_O, pD adjusted to 1.9 with diluted DCl, where “pD” is pH-meter readout uncorrected for isotopic effects, for 24 h at 65 °C either under quiescent conditions (in the case of of [BI] preparation) or intensive agitation at 1400 rpm using Eppendorf Thermomixer Comfort accessory (for [BI]^V^ preparation). For seeding experiments, mother [BI] fibrils were subjected to pulsed sonication using Ultrasonic Processor VC130PB from Sonics & Materials, Inc. (USA) operating at 20 kHz and 20% of nominal power of 130 watts. Sonication was carried out in intervals to ensure that amyloid samples are not excessively heated. Total sonication time was 60 s. Sonicated [BI] fibrils remained stable in solution and retained the catalytic activity (in terms of converting native insulin into amyloid) over the period of several weeks when stored at 4 °C, yet, only freshly sonicated fibrils were used in following experiments. An extensive characterization of morphological and spectral properties of sonicated seeds was carried out according to protocols established in previous studies^23^,^24^. Comparison of AFM images of different insulin fibrils before and after sonication has been placed in Supplementary Information.

For seeding experiments, sonicated mother fibrils were added to freshly prepared 1 wt. % insulin solutions in 0.1 M NaCl in D_2_O, pD 1.9 at 1:100 insulin fibrils: native insulin mass ratio. In order to prevent competitive *de novo* formation of amyloid nuclei temperature of the following incubation was set at 37 °C. Once formed, insoluble aggregates of daughter amyloid fibrils were subjected to morphological analysis. Prior to FT-IR measurements, or being used as seeds, daughter fibrils were sonicated, as specified above. It is well-established that seeding of insulin in the acidic environment and in the presence of NaCl with preformed amyloid templates leads to practically complete conversion of the native protein into fibrils^23^,^24^.

### FT-IR Spectroscopy

For FT-IR measurements, a CaF_2_ transmission cell equipped with a 0.05 mm Teflon spacer was used. Temperature in the cell was controlled through an external water-circuit connected to a programmable thermostat. All FT-IR spectra were collected on a Nicolet NEXUS FT-IR spectrometer equipped with a liquid nitrogen-cooled MCT detector. Typically, for a single spectrum 256 interferograms of 2 cm^−1^ resolution were co-added. During measurements, the sample chamber was continuously purged with dry CO_2_-depleted air. All insulin spectra were corrected by subtracting the proper amount of D_2_O and water vapor spectra prior to being baseline-adjusted. Data processing including calculations of second derivative spectra (Savitzky-Golay) was performed using GRAMS software (ThermoNicolet, USA). All further details have been described earlier^24^.

### Kinetic Measurements of ThT Fluorescence - Methodology

For kinetic experiments, Fluoroskan Ascent FL fluorometer equipped with a pair of λ_ex._ 440 nm/λ_em._ 485 nm optical filters and 96-well black microplates were used. Sample conditions were the same as for FT-IR measurements (1 wt. % BI in 0.1 M NaCl in D_2_O, pD adjusted to 1.9 with diluted DCl, with sonicated, as specified above, seeds added at a 1:100 insulin fibrils: native insulin mass ratio) except that ThT was added to the final concentration of 20 μM. Control experiments have shown that ±10 μM variation in ThT concentration does not affect significantly the appearances of kinetic trajectories. Kinetic experiments were carried out at 37 °C and gentle periodic agitation at 300 rpm. In order to assess reproducibility of aggregation kinetics, 6 microplate wells were filled with 170 μl portions of each sample for parallel measurements. All further details have been described earlier^24^.

### ICD Spectroscopy

75 μL portions of as-grown insulin fibrils in acidified D_2_O-based solutions were further diluted with 2 mL of 0.1 M NaCl in H_2_O, pH 1.9 containing 65 μM ThT. All ICD spectra were collected at 25 °C and under quiescent conditions on J-815 Spectropolarimeter from Jasco, Japan using 10 mm quartz cuvettes.

### AFM

Samples were diluted 60-times with deionized water. A small droplet (8 μl) of fibrils suspension was swiftly deposited onto freshly cleaved mica and left to dry overnight. AFM tapping-mode measurements were carried out using a Nanoscope III atomic force microscope from Veeco, U.S.A., and TAP300-Al sensors (res. frequency 300 kHz) from BudgetSensors, Bulgaria. Other experimental parameters were the same as in earlier studies^24^.

### Kinetic model of strain propagation

We have modelled the aggregation process following a simple model of polymerization as introduced by Collins *et al.*^50^. In the model, a set of coupled differential equation is introduced for the growth rates of populations of molecules of soluble native protein and amyloid fibrils of different lengths. Soluble protein can nucleate into amyloid (with the nuclei of size n, and nucleation rate of k_N_), whereas amyloid fibrils can elongate (with rate k_E_) or break into smaller pieces (with rate k_B_). An important difference with respect to the model of Collins and colleagues is the introduction of two different structural types of aggregates: A_1_ (corresponding to [BI] fibrils) and A_2_ ([BI]^V^-like amyloid) which can be independently created by nucleation of soluble protein. This requires introduction of two sets of kinetic parameters, one for each strain. We have used the following values: n_1_ = n_2_ = 4; k_N1_ = 5 * 10^−6^; k_N2_ = 3.09 * 10^−7^; k_E1_ = 0.80; k_E2_ = 0.96; k_B1_ = k_B2_ = 10^−9^. Another variable parameter in the model is “d” (defined as the power to which the rate of fibril fragmentation depends on its length) which was set uniformly to 3^48^. For a simulation of the *de novo* aggregation of agitated insulin samples we set the value k_B1_ = k_B2_ = 0.01. The resulting set of differential equations has been solved by a midpoint method. In the first cycle (*de novo* growth), we start from a pool of “soluble” insulin molecules. The polymerization begins with a slow nucleation phase, followed by a faster elongation phase accompanied by breaking of longer chains. The process is terminated when all available native insulin molecules are used up. The resulting amyloid populations are then diluted with the 1:100 dilution ratio and added to the next pool of the native insulin. In the successive cycles, nucleation processes are neglected (by setting k_N_ = 0).

## Additional Information

**How to cite this article**: Surmacz-Chwedoruk, W. *et al.* The emergence of superstructural order in insulin amyloid fibrils upon multiple rounds of self-seeding. *Sci. Rep.* **6**, 32022; doi: 10.1038/srep32022 (2016).

## Supplementary Material

Supplementary Information
