# Supplementary material for: The emergence of superstructural order in insulin amyloid fibrils upon multiple rounds of self-seeding
Source: Sci Rep. 2016 Aug 25;6:32022. doi: 10.1038/srep32022 (PMC4997315; doi:10.1038/srep32022)
Supplement: Supplementary Information [file srep32022-s1.pdf]

The emergence of superstructural order in insulin amyloid fibrils upon multiple rounds of self-seeding.

Weronika Surmacz-Chwedoruk, Viktoria Babenko, Robert Dec, Piotr Szymczak,

and Wojciech Dzwolak

Supplementary Information

1. **Effects of sonication on morphology of different insulin amyloid variants, as probed by AFM.**

2. **Simulation of impact of periodic sonication on spreading of conformational "mutations" in elongating amyloid fibrils.**

---

1. Effects of sonication on morphology of different insulin amyloid variants, as probed by AFM.

Various types of insulin amyloid fibrils prepared either by *de novo* aggregation or seeding, as described in the Materials and Methods section of the main article, were subjected to pulsed sonication using Ultrasonic Processor VC130PB from Sonics & Materials, Inc. (USA) operating at 20 kHz and 20 % of nominal power of 130 watts. The sonication was carried out in intervals to ensure that amyloid samples are not excessively heated. Total sonication time was 60s. Sonicated fibrils remained stable in solution and retained the catalytic activity (in terms of converting native insulin into amyloid) over the period of several weeks when stored at 4 °C. Samples of sonicated fibrils were diluted 60-times with deionized water.

A small droplet (8 μl) of fibrils suspension was swiftly deposited onto freshly cleaved mica and left to dry overnight. AFM tapping-mode measurements were carried out using a Nanoscope III atomic force microscope from Veeco, U.S.A., and TAP300-Al sensors (res. frequency 300 kHz) from BudgetSensors, Bulgaria. Other experimental details are placed in the Materials and Methods section of the main article or are referenced therein.

The following Figure SI_1 shows tapping mode AFM images of mother [BI], and $[BI]^V$ fibrils, as well as of selected (3rd, 6th and 12th) generations of daughter fibrils obtained through seeding before (left column) and after (right column) the sonication. The indicated scale bar is valid for all images.

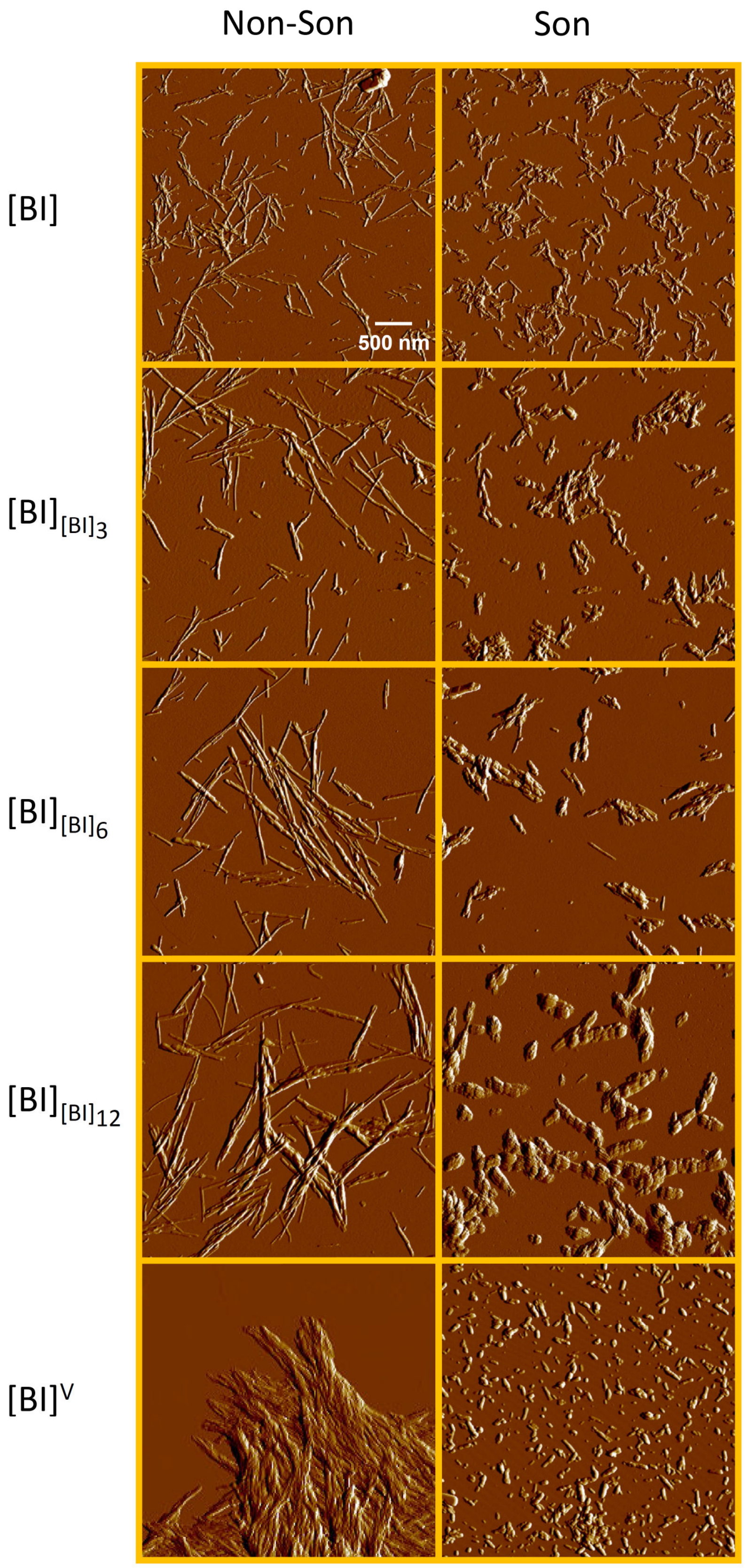

Figure SI_1. Effects of sonication on morphology of insulin fibrils.

While sonication clearly impacts all the insulin amyloid variants examined here, in each case resulting in breakage of fibrils into shorter scattered specimen, it is interesting to note that the identical regimes of ultrasound treatment lead to a more profound fragmentation of $[BI]^{V}$ fibrils than in the case of $[BI]_{12}$ amyloid.

---

2. Simulation of impact of periodic sonication on spreading of conformational "mutations" in elongating amyloid fibrils.

We have modelled possible impact of conformational switching (between two different amyloid conformers named "I" and "II") and periodic sonication of fibrils on proliferation of these two conformers. The results presented below provide an auxiliary argument against such a scenario in the case of the observed conformational drift reported in this study.

<u>Computational details:</u>

In the case of alternate elongation-and-division the mean numbers of monomers of both types (N_I and N_{II}) evolve in time according to the following difference equations:

$$N_I(t+1) = N_I(t) + N_I(t)(1 - P_{I\to II}) + N_{II}(t)P_{II\to I}$$
$$N_{II}(t+1) = N_{II}(t) + N_{II}(t)(1 - P_{II\to I}) + N_I(t)P_{I\to II}$$

with $N_I(0) = 1$and $N_{II}(0) = 0$. These equations can be solved to yield

$$N_I(t) = \frac{P_{I\to II}(2 - P_{I\to II} - P_{II\to I})^t + 2^t P_{II\to I}}{P_{I\to II} + P_{II\to I}} \qquad N_{II}(t) = \frac{P_{I\to II}(2^t - (2 - P_{I\to II} - P_{II\to I})^t)}{P_{I\to II} + P_{II\to I}}$$

The global excess of population I over II is then given by

$$\frac{N_I(t) - N_{II}(t)}{N_I(t) + N_{II}(t)} = -\frac{2^{1-n}P_{I\to II}(2 - P_{I\to II} - P_{II\to I})^t + P_{II\to I} - P_{I\to II}}{P_{I\to II} + P_{II\to I}}$$

In particular, for $P_{II\to I} = P_{I\to II}$the above reduces simply to

$$\frac{N_I(t) - N_{II}(t)}{N_I(t) + N_{II}(t)} = (1 - P_{I\to II})^t$$

whereas for $P_{II\to I} = 0$

$$\frac{N_I(t) - N_{II}(t)}{N_I(t) + N_{II}(t)} = 2^{1-t}(2 - P_{I\to II})^t - 1$$

The above formulas are used to plot the respective curves in Figure SI_2. Next, in the case of perpetual elongation, the state of the end monomer evolves according to:

$$p(t+1) = p(t) - P_{I\to II}p + P_{II\to I}(1 - p)$$

where $p(t)$ is the probability that the end monomer is in state I. Again, this can be solved to yield

$$p(t) = \frac{P_{I\to II}(1 - P_{I\to II} - P_{II\to I})^t + P_{II\to I}}{P_{I\to II} + P_{II\to I}}$$

The total number of monomers in state I can then be recovered as

$$N_I(t) = \sum_{i=0}^{t} p(i)$$

For a particular case of $P_{II\to I} = P_{I\to II}$the above leads to

$$\frac{N_I(t) - N_{II}(t)}{N_I(t) + N_{II}(t)} = \frac{(1 - 2P_{I\to II})^{1+t} - 1}{2P_{I\to II}(1 + t)}$$

whereas for $P_{II\to I} = 0$

$$\frac{N_I(t) - N_{II}(t)}{N_I(t) + N_{II}(t)} = \frac{2(1 - P_{I\to II})^{1+t} + P_{I\to II}(1 + t) - 2}{P_{I\to II}(1 + t)}$$

which was used to plot the respective curves in Figure SI_2.

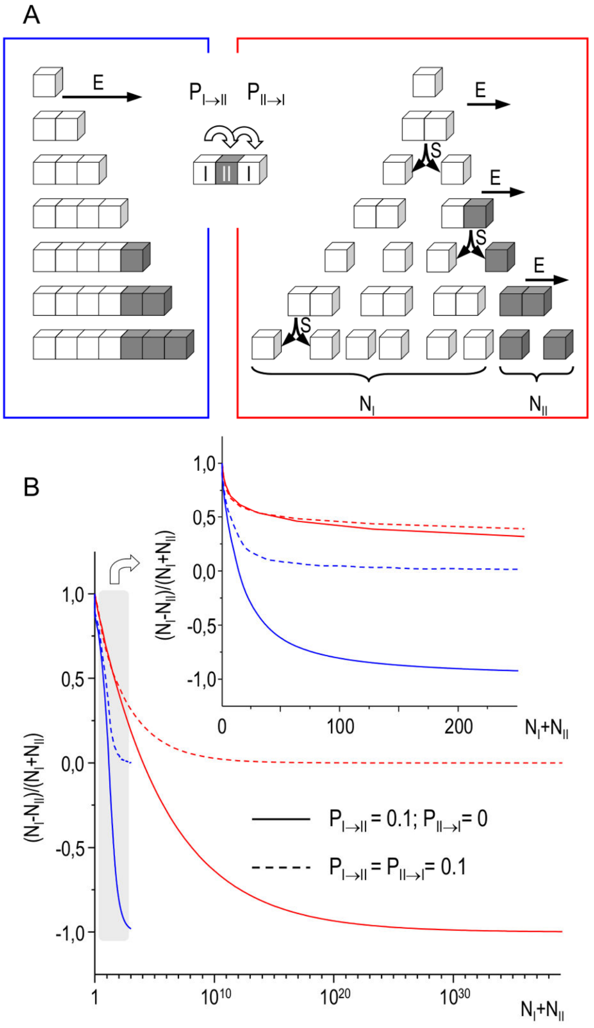

Figure SI_2. Simulated impact of periodic sonication on spreading conformational "mutations" in elongating amyloid fibrils. (a) Comparison of

propagation rates of two structural variants of fibrils (I and II) for two extreme amyloid growth scenarios (*perpetual elongation* – left blue panel, and *alternate elongation-and-division* – right red panel) in the context of sporadic conformational switching along the propagating fibril. Probability of a local switching event between two structural forms of fibrils (I and II) is $P_{I \to II}$. (b) Simulated averaged trajectories of the global excess of either structural variant plotted versus the total number of amyloid-converted monomers. Probability of structural mutation is arbitrarily set at 0.1. Perpetual elongation (blue lines) and alternate elongation-and-division (red lines) are considered for one-way (solid lines) and "reversible" (dashed) structural mutations. The dynamics of the initial changes are replotted on a linear N_I+N_{II} scale (inset).

Analysis:

The routine of repetitive sonication of all following generations of daughter fibrils prior to using them as seeds has two important consequences. Namely, it not only multiplies concentration of catalytically active tips, but is also expected to decrease any statistical impact of possible conformational switching events. For a perpetually elongating fibril built of type I blocks (Figure SI_2a), a spontaneous transition to an alternative type II at fibril's tip would effectively trap the type I elements preventing them from replicating (in the absence of other aggregation routes) thus giving an enormous proliferation advantage to the structural mutants of type II flanking fibrils' ends. Cyclic fragmentation of growing fibrils (e.g. through sonication) would release the trapped type I conformers giving them chance to spread their phenotype (Figure SI_2b). Hence, the repetitive fragmentation would have a dramatic impact on the rate of spreading of the two structural variants, which is illustrated by calculated idealistic trajectories of dominance of single amyloid variant for a set of arbitrary boundary conditions and the conformational switching event being either reversible or not.
